# 

**Published:** 1882-09

**Authors:** 


### CONTENTS:

PAGE.

ORIGINAL LECTURES:

I. Chronic General Diseases, or Constitutional Affections; their Causes, Pathology and the Principles that should guide their Treatment. By N. S. Davis, M.D. .......... 225

ORIGINAL COMMUNICATIONS:

II. Sewer Gas and Its Effects upon Health. By Byron W. Griffin, M.D. .......... 241

III. Amnesic Aphasia from Wound of Left Anterior Lobes of Cerebrum and Loss of Brain Substance. Partial Recovery. By E. Wyllys Andrews, M.D. .......... 250

SOCIETY REPORTS:

IV. Chicago Medical Society .......... 254

V. National Board of Health. Report of Inspector for Illinois District .......... 256

VI. American Public Health Association... 260

VII. Tri-State Medical Society .......... 261

FOREIGN CORRESPONDENCE:

VIII and IX. Paris Letters .......... 263

DOMESTIC CORRESPONDENCE:

X. A New Wrinkle in Hypodermic Medication .......... 271

XI. Is Yellow Fever Contagious? .......... 272

EDITORIAL:

Cook County Hospital .......... 274

BOOKS AND PAMPHLETS RECEIVED .......... 276

PAGE.

ORIGINAL TRANSLATIONS:

Extirpation of the Larynx .......... 278

Cancer of the Ileum .......... 280

ABSTRACTS:

Inhalation of Medicated Vapors in Diseases of the Respiratory Organs .......... 282

Insanity and Divorce .......... 284

Cool Air Etc., in Measles and Scarlet Fever. 285

Quinine in Cholera Infantum .......... 287

Geographical and Climatic Relations of Pneumonia .......... 289

SELECTIONS:

Report of Three Cases of Abscess of the Brain. By J. T. Eskridge, M.D. .......... 291

Bile and Digestion. By J. W. Stokes, M.D... 313

A Clinical Study of the Small Granular Cells of the Blood. By J. T. R. Davison, M.D. .......... 324

Syphilis: Its Relation to Scrofula. By John B. Roberts .......... 329

Tenosynovitis. By W. B. Hopkins, M.D. ..... 332

Threatened or Actual Epidemics .......... 334

ITEMS .......... 240, 262, 270, 273, 277, 281, 290

ANNOUNCEMENTS FOR THE MONTH .......... 336

NOTICE TO CORRESPONDENTS .......... *advts.*

